# Enhanced genome replication activity of pandemic H1N1 influenza A virus through PA mutations

**DOI:** 10.1128/jvi.01391-25

**Published:** 2025-12-23

**Authors:** Jordana Schmierer, Michael Lutz, Toru Takimoto

**Affiliations:** 1 Department of Microbiology and Immunology, University of Rochester Medical Center 6923, Rochester, New York, USA; Emory University School of Medicine, Atlanta, Georgia, USA

**Keywords:** influenza A virus, host adaptation, polymerase, replicase

## Abstract

The 2009 pandemic H1N1 (pH1N1) influenza A virus (IAV) is a reassortant virus with two polymerase components, PA and PB2, originating from avian IAV. Avian IAV polymerase does not function efficiently in mammalian cells without host-adaptive mutations. The mechanism by which pH1N1 replicates in human hosts is not fully elucidated, as pH1N1 does not contain the host-adaptive PB2 E627K mutation required for species-specific interaction with ANP32, which facilitates replicase (polymerase oligomer) formation. Our previous research revealed that mutations in PA played a key role in mammalian host adaptation of pH1N1. These mutations were found in two separate domains of PA, the C-terminal (CTD) and N-terminal domains (NTD). We reported that the NTD mutations increase the expression of NP through enhanced association of GRSF1 with the mRNA transcripts. However, the role of CTD mutations, which are located at the interface of the polymerase oligomers, has not been elucidated. In this study, we characterized the effect of key CTD mutations and found that the CTD mutations enhanced genome replication activity and replicase formation *in vitro*. Unexpectedly, rescued viruses containing only the CTD mutations that enhance genome replication activity had an attenuated viral growth phenotype. However, the introduction of an additional NTD mutation to the virus restored virus growth in mammalian cells. These results suggest that the mutations found in the PA NTD are required together with CTD mutations for balanced genome replication and growth in human cells.

**IMPORTANCE:**

The 2009 pandemic H1N1 (pH1N1) influenza A virus (IAV) is a reassortant virus with two polymerase components, PA and PB2, originating from avian IAV, which typically does not function well in mammalian cells. All the human IAVs, except pH1N1, contain E627K in the PB2 subunit, which allows the virus to utilize host factor ANP32 to form polymerase oligomers required for genome replication. The mechanism of how pH1N1 adapted to humans and caused seasonal epidemics is not yet fully elucidated, but our previous studies revealed that mutations in PA play a key role in host adaptation. Here, we describe a novel mechanism of host adaptation where mutations in the PA CTD enhance genome replication, whereas PA NTD mutations increase nucleoprotein production to support increased replication activity. This finding highlights the range of mechanisms of host adaptation, which is vital to understanding when assessing the potential emergence of novel viruses.

## INTRODUCTION

Seasonal outbreaks of influenza A virus (IAV) cause significant morbidity and mortality, and the virus has a high potential to emerge from animal reservoirs to cause future pandemics (1, 2). Therefore, understanding how IAV adapts to human hosts from its reservoir remains a priority. IAV adaptation can occur through reassortment of gene segments, leading to novel outbreaks, or through acquisition of specific host-adaptive mutations (3). It is well recognized that the viral RNA-dependent RNA polymerase (vRdRp) of avian IAV does not function well in mammalian cells (4, 5). The vRdRp is composed of subunits PB2, PB1, and PA. We and others have previously identified multiple host-adaptive mutations in the PB2 and PA components that allow avian IAVs to efficiently replicate and transmit in mammals (6–9). However, the mechanisms of how these mutations activate the avian IAV vRdRp have not been fully elucidated.

Recent advances in the structural analysis of the vRdRp revealed that the vRdRp forms oligomers for viral genome replication. Importantly, host protein acidic nuclear phosphoprotein 32 (ANP32) is required for vRdRp oligomer (replicase) formation, and the interaction between ANP32 and the vRdRp is species specific (10). The avian vRdRp requires the PB2 E627K mutation to interact well with human ANP32 in human cells, and hence, PB2 E627K has been recognized as a key mutation activating avian vRdRp in mammalian cells (11–13). ANP32 serves as a scaffold in the formation of vRdRp oligomers, and it recruits nucleoprotein (NP) to encapsulate the replicated RNA genome (14–16). A recent model of genome replication indicates that a newly synthesized vRdRp attaches to the vRdRp associated with the nucleocapsid and forms an asymmetric dimer together with ANP32. One of the vRdRps replicates the genome, and the other encapsidates the newly synthesized viral genome (17). In the asymmetric dimer, the PA C-terminal domain (CTD) of one vRdRp interacts with PB2 of the other vRdRp. This structure is proposed to be responsible for positive-sense cRNA synthesis from the negative-sense vRNA template. The current model also proposes that a third, *trans*-activating vRdRp associates with the asymmetric dimer bound to a nucleocapsid containing the positive-sense cRNA. This association realigns the cRNA template for subsequent vRNA replication (18, 19). The PA CTD of the third vRdRp interacts with the PA CTD of the replicating vRdRp, creating a symmetric interaction (20).

As explained above, recruitment of human ANP32 through PB2 E627K is vital for host adaptation of avian IAV to infect humans. All the past pandemic IAVs contained the PB2 E627K mutation except for the 2009 pandemic H1N1 (pH1N1) (21). The pH1N1 is a reassortant virus that emerged from the swine reservoir and has two polymerase components, PA and PB2, originating from avian IAV (22, 23). Mutations in both PA and PB2 have been identified to enhance avian vRdRp in mammalian cells in the absence of PB2 627K. pH1N1 PB2 residues 590/591 have been shown to enhance vRdRp activity, and recent research has revealed that PB2 591 specifically allows the vRdRp to recruit mammalian ANP32 (24, 25). Additionally, previous work in our lab identified mutations in PA that play a key role in enhancing vRdRp activity in mammalian cells (6, 7, 26). Using a luciferase reporter assay, we reported that two mutations present in the PA N-terminal domain (NTD), T85I and G186S, and one in the PA CTD, L336M, of 2009 pH1N1 significantly enhanced vRdRp activity (7). Recently, we found that PA NTD mutations enhance the expression of viral NP by increasing the attachment of cellular RNA-binding protein GRSF1 to NP mRNA (6). We demonstrated a link between PA NTD mutations and host factor GRSF1 in facilitating the efficient cytosolic accumulation and translation of viral mRNAs, which has important consequences for viral fitness. Interestingly, the pH1N1 had acquired further mutations in both the PA NTD and CTD during seasonal circulation. One of these mutations at PA residue 321 was shown to enhance vRdRp activity, suggesting a role in adaptation to human hosts (27).

Currently, the role of PA CTD mutations in host adaptation is not fully elucidated. However, the CTD mutations found in pH1N1 viruses are located at the interfaces of both the symmetric and asymmetric vRdRp dimers (17, 20). Therefore, we tested if PA CTD mutations found in pH1N1 viruses enhance vRdRp oligomer formation and accelerate genome replication without PB2 E627K. Our analysis using *in vitro* reporter gene assays showed that the CTD mutations indeed affect vRdRp oligomer formation and enhance genome replication activity. However, characterization of rescued mutant viruses showed that the PA CTD mutations alone are detrimental for virus growth without enhanced NP expression regulated by PA NTD mutations. This study indicates that the 2009 pH1N1 virus adapted to human hosts through PA CTD and NTD mutations that together enhance genome replication activity and rapid spread.

## RESULTS

### Comparison of polymerase activity between avian IAV and pH1N1 isolates

Previous work in our lab showed that although pH1N1 PB2 partially enhances the avian IAV vRdRp, PA has the greatest effect on enhancing the activity of an otherwise avian vRdRp in mammalian cells (6, 7, 26). Sequence data indicate that there are 19 residues that differ in PA between a representative avian IAV A/chicken/Nanchang/3-120/01 (Nan) and A/California/04/2009 (Cal), and 30 residues that differ in PB2 between Nan and Cal (Fig. S1a). As pH1N1 continued circulating seasonally, the virus has acquired additional mutations in PA and PB2, which may further enhance viral fitness to human hosts. The additional mutations of the 2017 isolate A/Michigan/272/2017 (Mich) include six residues in PA and 11 residues in PB2 (Fig. S1b).

**Fig 1:**
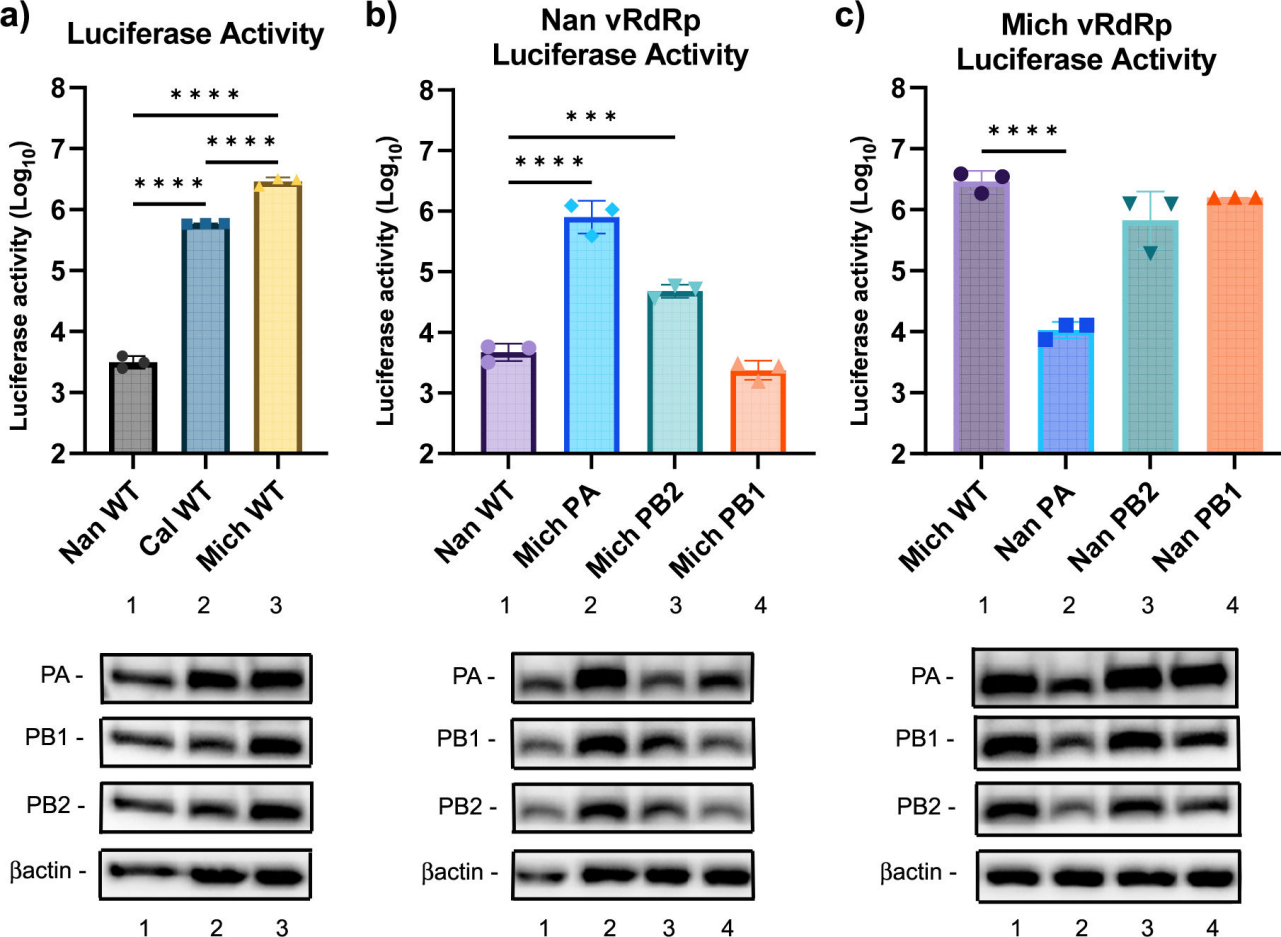
Characterization of vRdRp activities. 293T cells were transfected with PA, PB1, PB2, and NP cDNAs from the indicated viruses together with the reporter cDNA for 24 h. Luciferase activity was measured by the Dual-Luciferase Reporter Assay system (Promega). (**a**) Activity of the RdRp complex of the three viruses. (**b**) Activity of Nan vRdRp with the indicated subunit replaced with that of Mich. (**c**) Activity of Mich vRdRp with the indicated subunit replaced with that of Nan. Expression of polymerase proteins was analyzed by western blotting. All error bars show means ± the standard deviations. *N* = 3 biological replicates. One-way ANOVA followed by Tukey’s multiple comparison test (***P < 0.001, ****P < 0.0001).

To analyze the role of each polymerase protein in host adaptation, we first compared the polymerase activity of Nan, Cal, and Mich using a standard reporter gene assay. Our luciferase reporter cDNA includes the negative-sense luciferase gene flanked by the 5′ and 3′ UTR of the IAV NP gene, which was transfected into HEK-293T cells along with various combinations of cDNA of the vRdRp subunits from either Nan, Cal, or Mich, and luciferase activity was quantified (Fig. 1a). The 2009 Cal vRdRp and 2017 Mich vRdRp produced about 200-fold to 900-fold more luciferase relative to the Nan vRdRp. Also, 2017 Mich vRdRp produced more luciferase than 2009 Cal vRdRp, which reflects the enhanced translation efficiency of mRNAs through additional Mich PA NTD mutations (6).

We next determined whether PA, PB1, or PB2 from Mich enhance avian Nan vRdRp activity. 293T cells were transfected with components of the Nan vRdRp, replacing a single subunit at a time with the corresponding Mich subunit, and quantities of the luciferase activity were measured (Fig. 1b). In agreement with our previous work with 2009 Cal, replacing PA had the greatest effect on luciferase production by the Nan vRdRp. Replacing PB2 also enhanced luciferase production, although to a lesser extent than PA. Replacing PB1 did not affect vRdRp activity. We also tested whether replacing each Mich vRdRp subunit with the corresponding Nan subunits would attenuate vRdRp activity (Fig. 1c). As expected, replacing PA with Nan PA significantly attenuated luciferase production relative to the Mich WT vRdRp. In contrast, replacing PB2 or PB1 did not significantly attenuate vRdRp activity. These data indicate that PA from both 2009 and 2017 pH1N1 isolates most strongly enhances vRdRp activity of avian IAV.

### Optimization of reporter assay to quantify genome replication

Given our recent finding that PA mutations affect the translational efficiency of viral mRNAs, the reporter gene assay measuring the luciferase activity may not accurately reflect the genome replication activity of vRdRp (6). Therefore, we directly quantified the replicated vRNA or cRNA in transfected cells using strand-specific qRT-PCR (28). The luciferase reporter cDNA produces negative-sense vRNA from which we can measure cRNA production. We produced a second, positive-sense NPLuc reporter cDNA in pPolI with the orientation of the Luciferase gene flipped to serve as a cRNA template to measure vRNA production (Fig. S2a). 293T cells were transfected with cDNAs expressing a functional Cal vRdRp or a nonfunctional Cal vRdRp lacking PA with either the negative- or positive-sense NPLuc reporter cDNA. cRNA from the negative-sense reporter and vRNA from the positive-sense reporter were quantified (Fig. S2b). We detected 20-fold or 90-fold more cRNA or vRNA production by vRdRp, compared with a nonfunctional vRdRp lacking PA. The vRdRp produced higher levels of vRNA from the positive-sense reporter RNA relative to cRNA production from the negative-sense reporter, which is consistent with previous findings showing that cRNA to vRNA replication is more efficient in viral infection (29).

**Fig 2:**
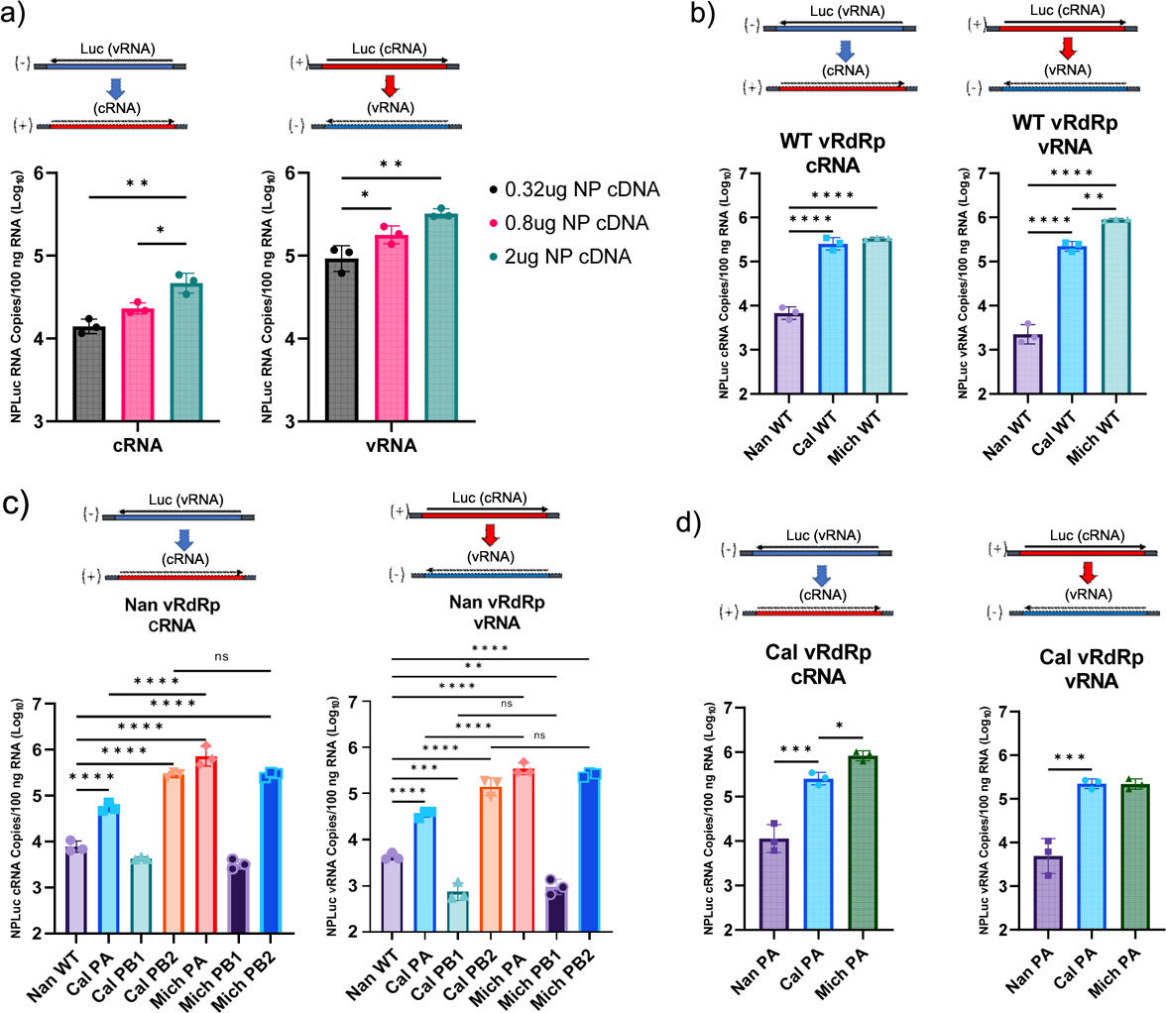
Origin of PA affects replicase activity. (**a**) 293T cells were transfected with 40 ng each Cal PA, PB1, and PB2, the indicated NPLuc reporter and varying amounts of Cal NP cDNA. Luciferase mRNA, cRNA, and vRNA were quantified by strand-specific qRT-PCR. (**b**) 293T cells were transfected with PA, PB2, PB1, and NP from the indicated virus and either negative- or positive-sense luciferase reporter cDNA. (**c**) 293T cells were transfected with the reporter genes, vRdRp and NP cDNAs from avian Nan virus, whereas the identified subunit was replaced with the corresponding Cal or Mich cDNAs. (**d**) 293T cells were transfected with reporter genes, PB2, PB1, and NP cDNAs from Cal virus and indicated PA cDNA. Luciferase cRNA or vRNA was quantified by strand-specific qRT-PCR. All error bars show means ± the standard deviations. *N* = 3 biological replicates. Statistical analysis was conducted using one-way ANOVA followed by Tukey’s multiple comparison test. ns, *P* > 0.05; *, *P* < 0.05; **, *P* < 0.01; ***, *P* < 0.001; ****, *P* < 0.0001.

We also optimized for the condition of genome replication by adjusting the quantities of NP provided in the cells, since a large amount of NP is required to encapsulate the replicated genome. 293T cells were transfected with a fixed amount of cDNAs for components of the Cal vRdRp and either the negative- or positive-sense reporters, together with a variable dose of NP cDNA. The quantities of replicated cRNA or vRNA were determined using strand-specific qRT-PCR (Fig. 2a). Transfection of 2 μg of NP cDNAs with 40 ng each of PA, PB1, and PB2 cDNAs produced the highest amount of replicated cRNA or vRNA, showing the importance of balance between the proteins needed for genome replication activity.

### Origin of PA determines replicase activity

Using the reporter assay optimized for replication, we next tested the genome replication activity of each vRdRp. 293T cells were transfected with all components from the Nan, Cal, or Mich vRdRp and either the negative- or positive-sense reporter (Fig. 2b). As expected, both the Cal and Mich vRdRps showed higher cRNA and vRNA production than the Nan vRdRp. Interestingly, when comparing genome replication activity between the Cal and Mich vRdRps, the Mich vRdRp produced more vRNA from the cRNA template than the Cal vRdRp, and this difference was not detected when comparing cRNA production from the vRNA template. These results may suggest that the Mich vRdRp contains mutations in its polymerase genes to specifically enhance (+) to (−) sense genome replication.

We next evaluated the impact of each component of the vRdRp on the genome replication activity of the avian (Nan) vRdRp (Fig. 2c). Replacing the PA of the Nan vRdRp with that of Cal PA enhanced genome replication activity from both the negative- and positive-sense templates. Interestingly, Mich PA further enhanced the activity of Nan vRdRp, suggesting that additional residues introduced in PA during seasonal circulation enhance the genome replication activity of the avian vRdRp. In addition, Cal and Mich PB2 enhanced genome replication, which is expected, as PB2 is also involved in the vRdRp dimer (replicase) formation and contains additional host-adaptive mutations (30–32). However, the genome replication activities of Nan vRdRp containing either Cal or Mich PB2 were similar, unlike those containing Cal or Mich PA. Cal and Mich PB1 attenuated genome replication, likely due to the mismatch in the interaction between Nan and Mich subunits that lowers overall activity (33).

We also tested genome replication activity with the backbone of the Cal vRdRp (Fig. 2d). Introduction of the Nan PA into the Cal vRdRp significantly attenuated replication activity from both templates as anticipated. In contrast, the introduction of the Mich PA enhanced cRNA production from the vRNA template, although there was no significant difference in vRNA synthesis from cRNA. These data indicate that human-adapted pH1N1 PA is a major factor in increased replication activity and that PA from seasonally circulating pH1N1 acquired mutations to further enhance replication activity.

### Impact of each host-adaptive mutation on the genome replication activity of an avian Nan vRdRp

**Fig 3:**
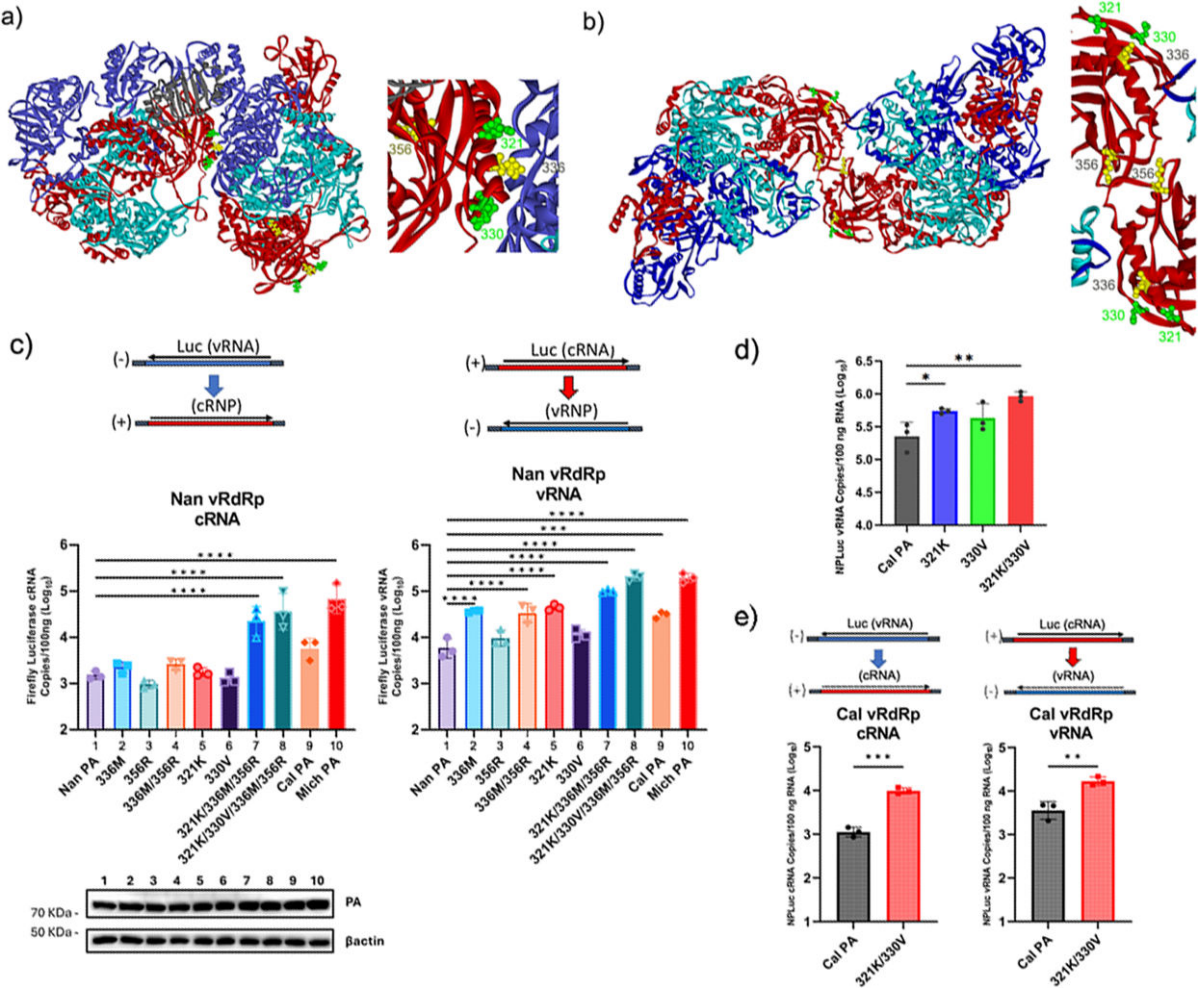
Effect of PA host-adaptive mutations on genome replication. (**a**) (left) Cryo-EM structure of asymmetric dimer of avian H5N1 RdRp in complex with ANP32 (PDB: 8R1J). PA, PB1, PB2, and ANP32B are colored in red, light blue, dark blue, and gray, respectively. The host-adaptive mutations in 2009 Cal, 336, and 356 and additional mutations in 2017 Mich, 321, and 330 are highlighted in yellow and green, respectively. (Right) Magnified view of asymmetric dimer interface with host-adaptive mutations labeled in yellow and green. (**b**) Structure of symmetric dimer of avian H5N1 RdRp (PDB 6QPF). Molecules and residues are colored as (**a**). (**c**) 293T cells were transfected with Nan PB1, PB2, and NP, indicated PA and luciferase reporter cDNA. Luciferase cRNA from the negative-sense reporter and vRNA from the positive-sense reporter were quantified by strand-specific qRT-PCR at 16 h post-transfection. Expression of the PA mutants was analyzed by western blotting. (**d**) 293T cells were transfected with Cal PB1, PB2, and NP, indicated PA and Luciferase negative-sense reporter cDNA. Luciferase cRNA was quantified by strand-specific qRT-PCR at 16 h post-transfection. (**e**) 293T cells were transfected with Cal PB1, PB2, and NP, indicated PA and Luciferase reporter cDNAs. Luciferase cRNA or vRNA was quantified by strand-specific qRT-PCR at 8 h post-transfection. All error bars show means ± the standard deviations. *N* = 3 biological replicates. Statistical analyses were conducted using one-way ANOVA followed by Tukey’s multiple comparison test (*P < 0.05, **P < 0.01, ***P < 0.001, ****P < 0.0001).

We next determined the impact of each PA CTD mutation on enhanced replication activity. Recent structural analysis indicates that the IAV vRdRp can form an asymmetric dimer in complex with cellular ANP32, which is required for (−) to (+) genome replication. Attachment of an additional vRdRp through a symmetric interaction with this dimer is considered to be required for the switch to (+) to (−) genome replication (18, 34). Our lab previously reported that the 2009 Cal PA CTD L336M mutation significantly enhances the replication of a virus containing the avian vRdRp in human cells and pathogenicity in mice (7). The other 2009 Cal PA CTD mutation K356R locates close to the residue 336 and was reported to enhance vRdRp activity (9). In addition, the PA of the 2017 pH1N1 isolate Mich includes two more mutations around this region at N321K and I330V, which were gained during pH1N1 seasonal circulation (Fig. S1b). The N321K was found in the majority of pH1N1 isolates by 2011, and I330V was found in the majority of pH1N1 isolates by 2014. Both residues have been maintained in the virus population since 2011 and 2014, respectively (Fig. S3). As seen in the cryo-EM structure, three of the four residues (321, 330, and 336) are located near the interface of the asymmetric dimer (Fig. 3a). In the symmetric dimer, residue 356 is located at the interacting loops between the two polymerase complexes, whereas the other three residues are surface exposed near the interface (Fig. 3b).

We therefore tested the impact of these CTD residues on genome replication activity. Mutations were introduced into Nan PA individually or in combination. 293T cells were transfected with cDNAs expressing Nan NP, PB1, and PB2 along with the indicated mutant PA and luciferase reporter cDNA. Quantities of synthesized cRNA from vRNA template, as well as vRNA from cRNA templates, were determined by strand-specific qRT-PCR (Fig. 3c). No individual mutation increased cRNA synthesis from the vRNA template. However, the combination of all four residues strongly enhanced cRNA production to a similar level as the full Mich PA subunit and greater than the full Cal PA subunit. For vRNA synthesis from the cRNA template, a single mutation at either L336M or N321K increased vRNA production by the avian vRdRp. Again, the mutant PA containing all four residues had the strongest activity and had similar activity to a vRdRp containing the full Mich PA subunit. These results indicate that residues located at or close to the vRdRp oligomer interfaces significantly affect the genome replication activity of avian (Nan) vRdRp. The mutations affected negative to positive replication differently than positive to negative, suggesting that they may have different roles in the PA-PB2 interface of the asymmetric dimer compared with the PA-PA interface of the symmetric dimer.

We next determined whether the two PA CTD mutations introduced during seasonal circulation increased replication activity of the 2009 Cal vRdRp. Residues 321 and 330 in Cal PA were mutated to lysine and valine, respectively. 293T cells were transfected with cDNAs expressing Cal NP, PB1, PB2, and the Cal PA wt, N321K, I330V, or N321K/I330V, along with the (−) luciferase reporter. Introduction of the single mutation N321K, but not the I330V mutation, significantly enhanced cRNA production from vRNA (Fig. 3d). However, when tested in combination, the I330V mutation further enhanced the genome replication activity of the N321K mutant. This enhanced activity was also detected for cRNA to vRNA replication (Fig. 3e). These results demonstrate that host-adaptive mutations acquired during seasonal circulation further enhance replication activity of a pH1N1 vRdRp.

### Effect of PA mutations on oligomer formation of Cal and Nan vRdRps

**Fig 4:**
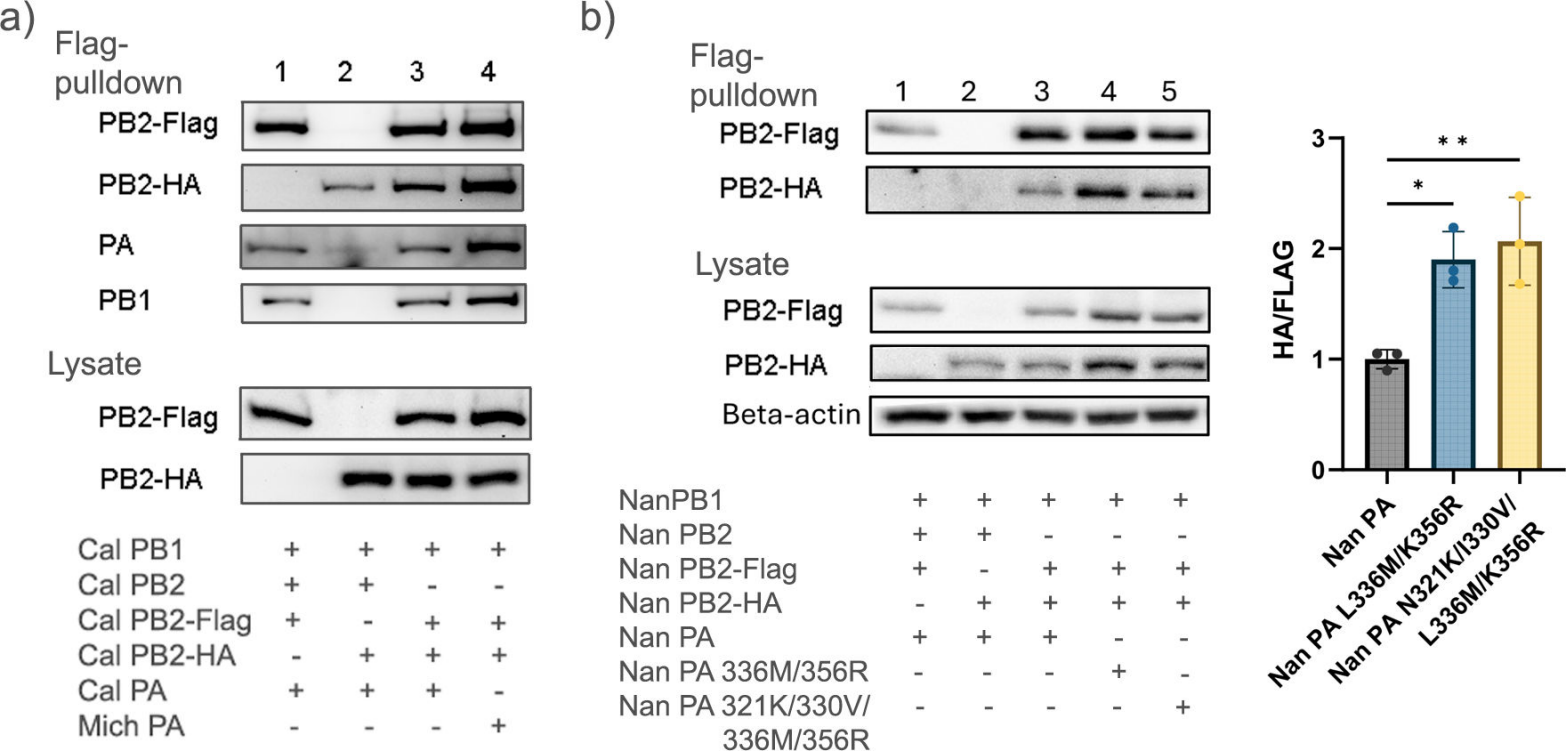
Effect of PA on vRdRp oligomer formation. (**a**) 293T cells were transfected with cDNAs expressing Cal PB1, Flag- or HA-tagged Cal PB2, and indicated PA. Anti-FLAG Ab was used for immunoprecipitation, and pulled down RdRp components were detected by specific Abs. The RdRps expressed in the cells were detected by specific Abs, as well. (**b**) 293T cells were transfected with Nan PB1, Flag- or HA-tagged Nan PB2, and wt or mutant Nan PAs. Anti-FLAG Ab was used for immunoprecipitation. Representative images of immunoblot analysis of recovered proteins and in whole cell lysate are shown. The quantity of HA- and FLAG-tagged PB2 was calculated by densitometry analysis, and the PB2-HA level was normalized to PB2-FLAG. All error bars show means ± the standard deviations. *N* = 3 biological replicates. Statistical analysis was conducted using one-way ANOVA followed by Tukey’s multiple comparison test in PRISM (*P < 0.05, **P < 0.01).

Given the location of our CTD mutations of interest, we next determined if these residues directly affect oligomer formation of the vRdRp by co-immunoprecipitation assay. We added a Flag- or HA-tag at the C-terminal of PB2, which will not interfere with asymmetric or symmetric dimer formation (Fig. S4). First, we determined if mutations in Mich PA affect the oligomer formation of Cal vRdRp. Cell lysates expressing Cal vRdRps containing Flag- or HA-tagged Cal PB2 and Cal or Mich PA were separately prepared, so that an equal amount of Flag- and HA-tagged vRdRps can be applied to the reaction mixture. The Flag-tagged and HA-tagged lysates were mixed and reacted with Protein G-Dynabeads conjugated with anti-Flag Ab overnight. After washing, the pulldown materials were analyzed by western blot analysis. Although Cal PB2-HA slightly cross-reacted with anti-Flag Ab, much more Cal PB2-HA was co-immunoprecipitated when mixed with vRdRp containing Cal PB2-Flag, indicating that Cal vRdRp forms oligomers (Fig. 4a, lane 3). Importantly, more oligomers were pulled down with vRdRp containing Mich PA than Cal PA (lane 4), suggesting that additional mutations introduced in PA during seasonal circulation further enhance the oligomer formation of pH1N1 vRdRp.

**Fig 5:**
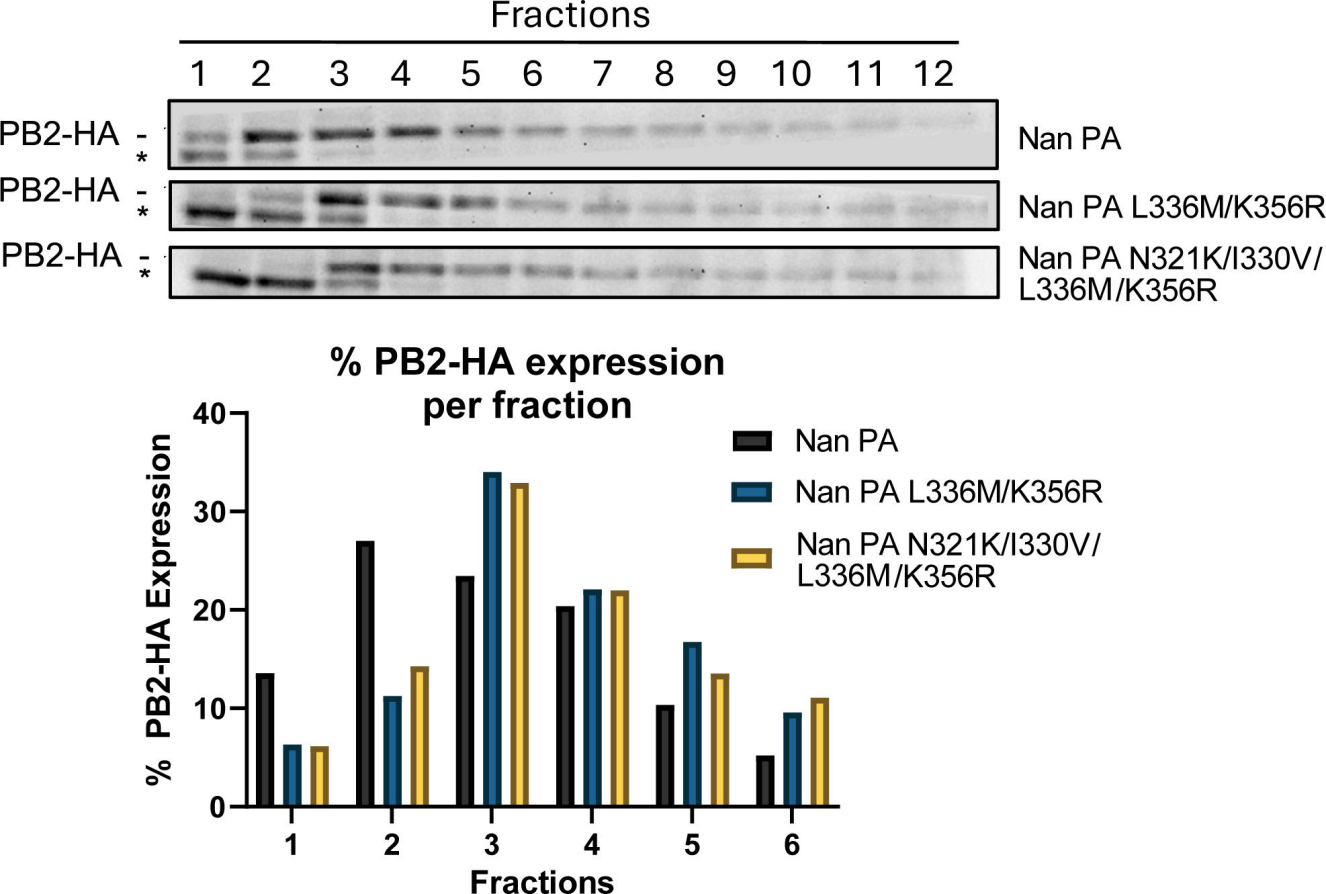
Effect of PA CTD mutations on vRdRp oligomer formation. (Top panel) 293T cells were transfected with cDNAs expressing Nan PB1, FLAG- or HA-tagged Nan PB2, and indicated PA. Total cell lysates were fractionated by sucrose gradient, and proteins in each fraction were detected by western blot analysis using anti-HA Ab. The top band is PB2-HA and was quantified using ImageJ software. The bottom band (*) is a nonspecific cellular protein reacting with the HA Ab. (Bottom panel) Quantities of PB2-HA in each fraction were measured, and the percentage of the proteins in each fraction relative to total protein quantity was calculated and shown in the bottom.

We further tested whether mutations L336M/K356R from 2009 Cal or combined mutations N321K/I330V/L336M/K356R in 2017 Mich could enhance dimer formation of an avian Nan vRdRp. 293T cells were transfected with Nan PB1, Flag- or HA-tagged Nan PB2, and the wt or mutant Nan PAs, and the samples were processed for immunoprecipitation. In the immunoprecipitated samples, the wt Nan vRdRp showed the lowest oligomer formation, whereas the addition of either the two Cal residues alone or all four adaptive residues significantly enhanced vRdRp oligomer formation (Fig. 4b). To further confirm the results of the pull-down assay described above, we measured vRdRp oligomer formation by density gradient centrifugation. The cells were transfected with Nan PB1, Flag- and HA-tagged Nan PB2, and the indicated Nan PA. We applied cell lysates over a 10%–40% sucrose gradient and centrifuged. After fractionation, the vRdRp proteins in each fraction were detected by western blot analysis. Comparison of the protein distribution across fractions clearly indicates a shift of more HA-tagged PB2 protein in lower fractions with a vRdRp containing either the two or four PA mutations (Fig. 5). Overall, our results suggest that either the Cal residues L336M/K356R or the Cal and Mich residues in combination N321K/I330V/L336M/K356R can enhance dimer formation of an avian vRdRp.

### Effect of CTD mutations on vRdRp activities in infected cells

**Fig 6:**
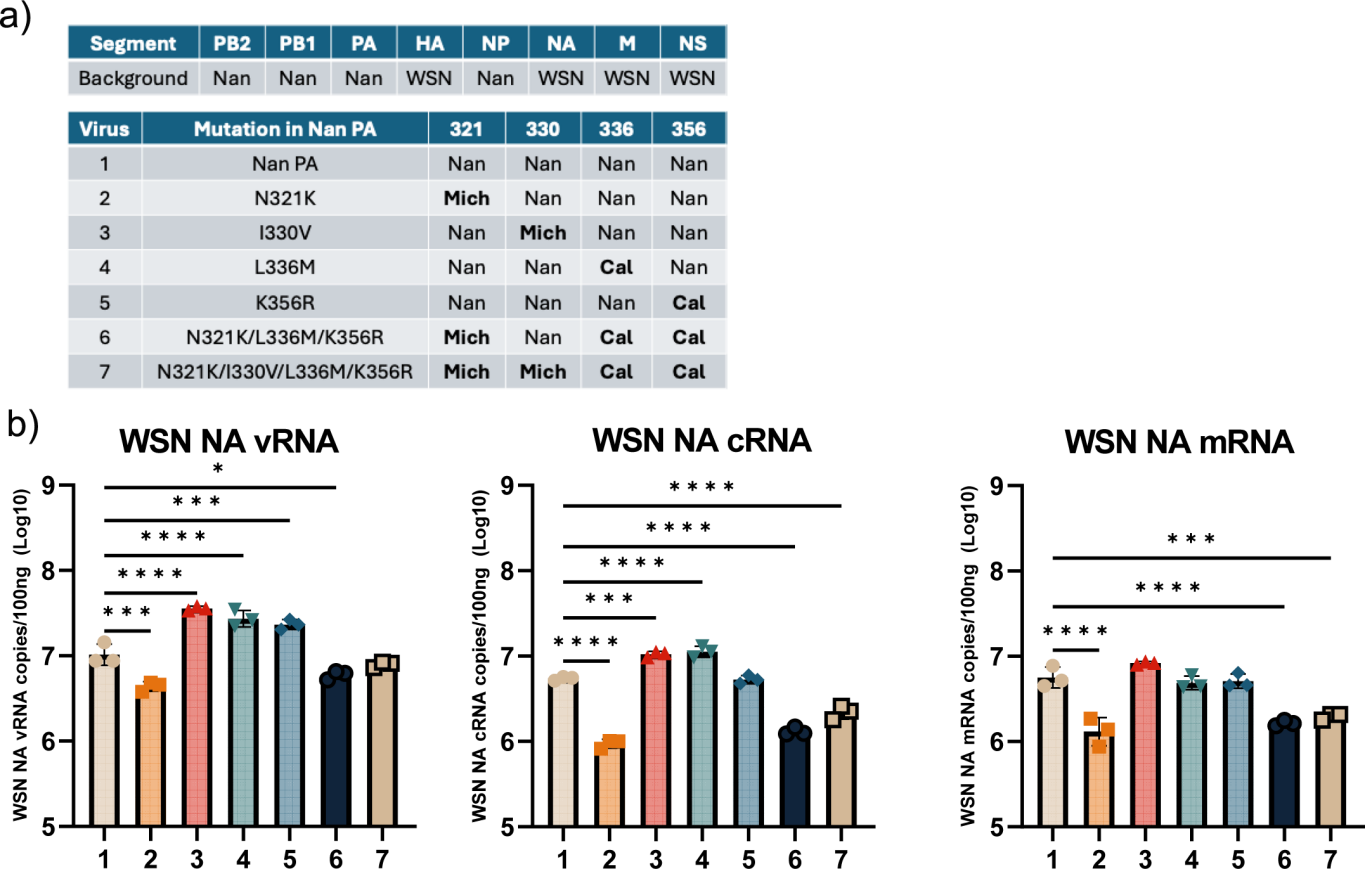
Genome replication and transcription by mutant viruses in infected cells. (**a**) The background of viral segments and the amino acid identity of each residue in each virus. (**b**) A549 cells were infected with the indicated viruses at an MOI of 2. At 16 hpi, RNA was extracted, and vRNA, cRNA, and mRNA of the NA gene were determined by strand-specific qRT-PCR. All error bars show means ± the standard deviations. *N* = 3 biological replicates. Statistical analyses were conducted using one-way ANOVA followed by Tukey’s multiple comparison test (*P < 0.05, ***P < 0.001, ****P < 0.0001).

To determine if the PA CTD mutations that enhance avian IAV vRdRp activity *in vitro* are sufficient to enhance genome replication in the context of virus infection, we rescued chimeric viruses containing Nan PB2, PB1, NP, and wt or mutant PA gene segments and the rest of the gene segments from A/WSN/1933 (H1N1) (WSN). These chimeric viruses allow us to directly assess the impact of mutations in avian virus vRdRp in infected cells. Key Nan PA CTD residues were mutated to those of Cal or Mich either individually or in combination, as done for *in vitro* analysis (Fig. 6a). Human alveolar epithelial A549 cells were infected with each virus, and synthesis of vRNA, cRNA, and mRNA of NA segment was determined at 16 h post-infection (hpi) (Fig. 6b). Individual mutation at residues 330, 336, and 356 slightly enhanced production of vRNA. However, a single mutation at residue 321 resulted in attenuated vRdRp activity in contrast to the results of the reporter gene assay, which showed enhanced genome replication activity (Fig. 3c). Similarly, the combined four mutations, which strongly enhanced genome replication activity *in vitro*, did not increase vRdRp activity in virus-infected cells. These results suggest that PA CTD mutations by themselves are not sufficient to enhance genome replication in the context of viral infection.

**Fig 7:**
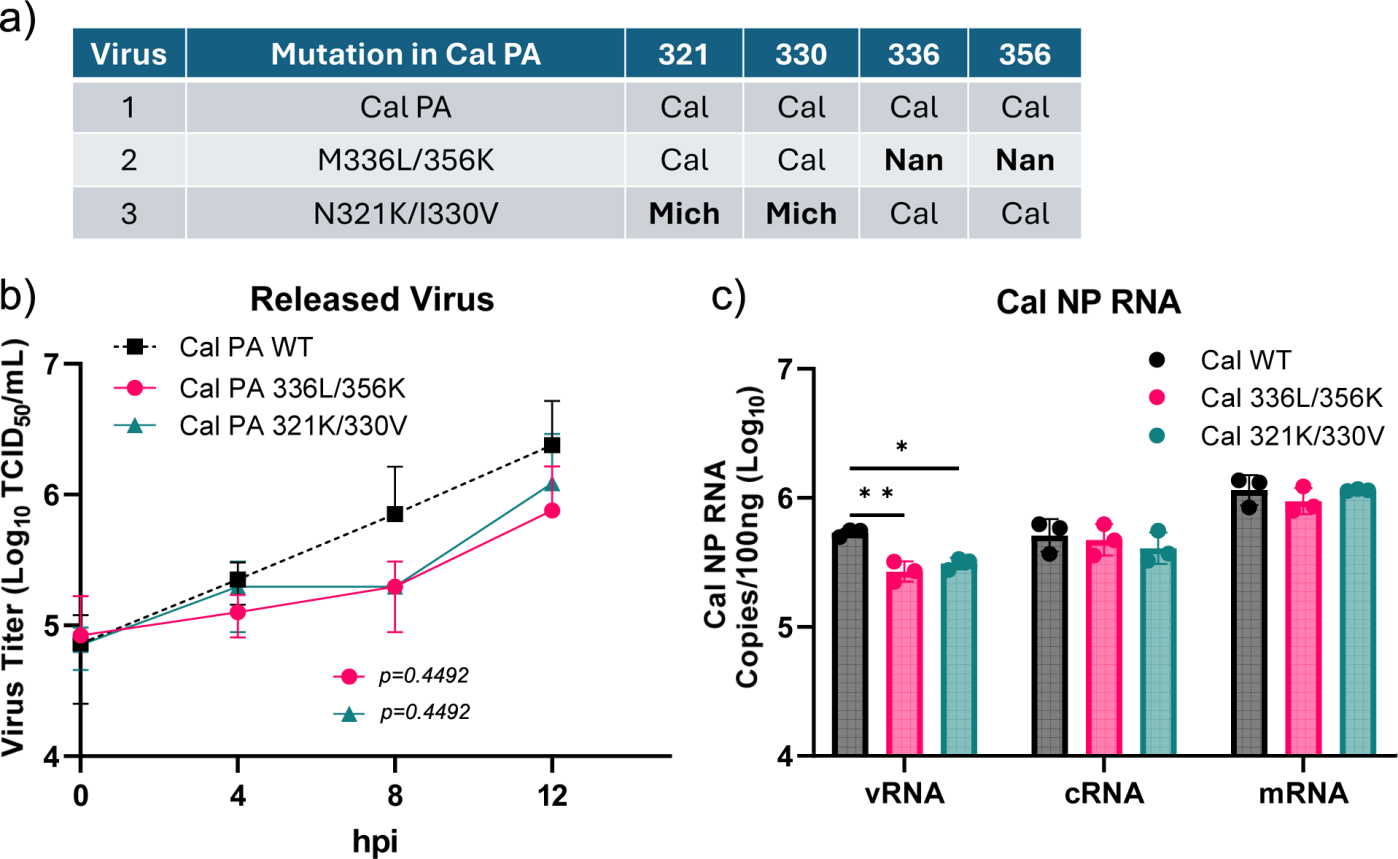
Effect of PA CTD mutations on growth and genome replication of 2009 pH1N1 virus. (**a**) Table indicates the amino acid identity of each residue in each virus. (**b**) A549 cells were infected with the indicated viruses at an MOI of 3 and cultured in the medium without trypsin to measure a single cycle replication. At indicated times, virus supernatant was collected and titrated after adding acetylated trypsin at 2 μg/mL. *N* = 3 biological replicates. (**c**) A549 cells were infected with the indicated viruses at an MOI of 2. At 8 hpi, RNA was extracted, and vRNA, cRNA, and mRNA of the NP gene were determined by strand-specific qRT-PCR. All error bars show means ± the standard deviations. *N* = 3 biological replicates. Two-way ANOVA followed by Tukey’s multiple comparison test (*P < 0.05, **P < 0.01).

We further analyzed the effect of PA CTD mutations using pH1N1 viruses. We rescued Cal viruses that contained the attenuating avian virus residues M336L/R356K or enhancing Mich residues N321K/I330V in PA (Fig. 7a). A549 cells were infected with each virus, and single-cycle viral growth was analyzed by quantitating infectious progeny virions (Fig. 7b). We also quantified vRNA, cRNA, and mRNA of NP gene in cells collected at 8 hpi (Fig. 7c). The virus with avian residues M336L/R356K had an attenuated growth phenotype, as expected. Also, the vRNA level in infected cells was significantly lower than Cal wt. The virus with Mich CTD residues N321K/I330V was also attenuated in virus growth in A549 cells and produced less vRNA. Together with the results of WSN/Nan chimeric viruses (Fig. 6), our data indicate that host-adaptive mutations in the PA CTD, which enhance genome replication activity, are detrimental for virus growth by themselves. These data may suggest a requirement of additional mutations in Cal or Mich, possibly those found in the PA NTD that enhance NP production, for supporting genome replication activity by PA CTD mutations to ultimately lead to enhanced virus replication.

### Increased NP production is required to enhance genome replication

**Fig 8:**
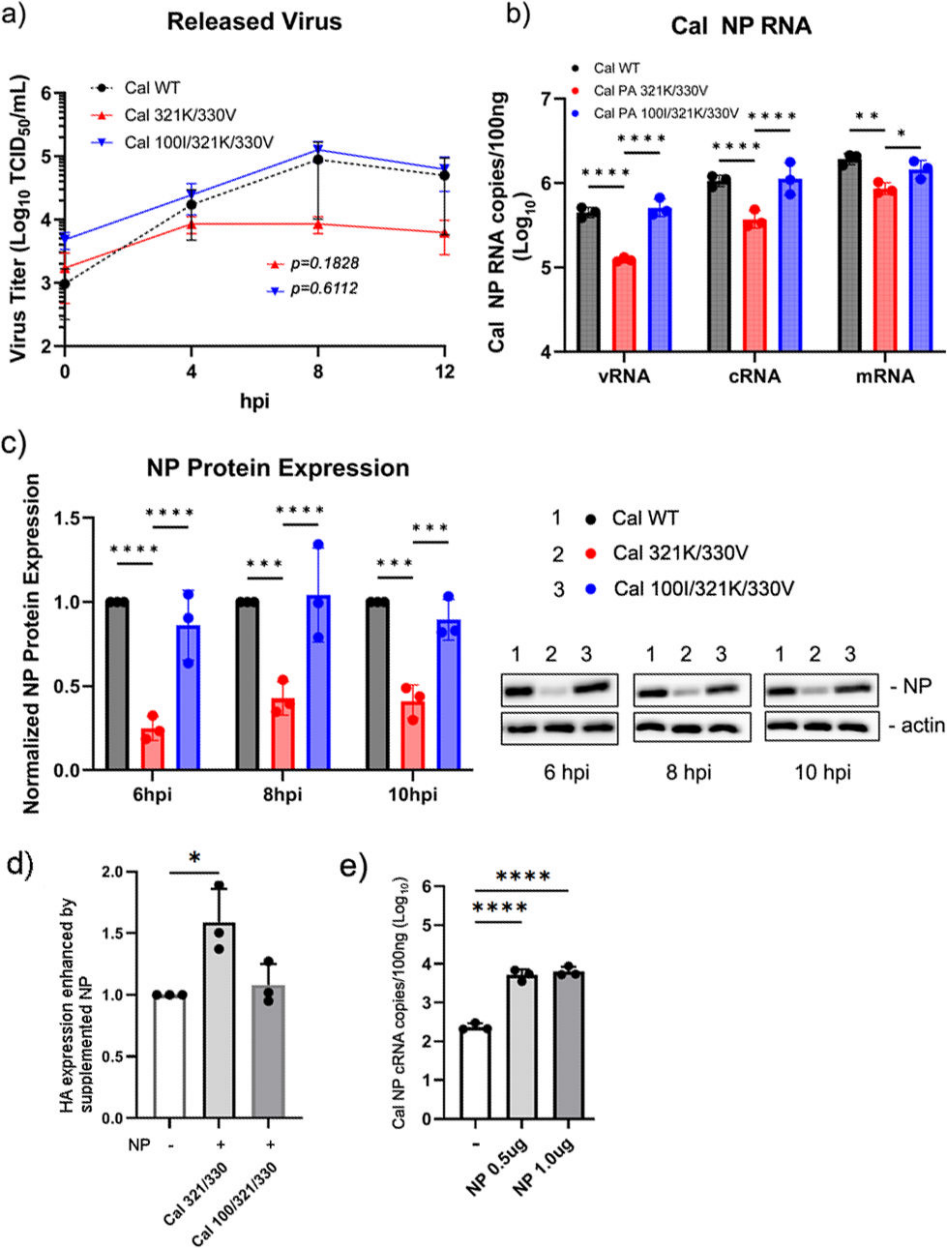
Mutations in both PA NTD and CTD are required to enhance viral growth and genome replication. (**a**) A549 cells were infected with the indicated viruses at an MOI of 0.2 and cultured in the medium without trypsin to measure single cycle replication. At indicated times, the virus supernatant was collected and titrated by measuring TCID_50_ in MDCK cells after adding acetylated trypsin at 2 μg/mL. (**b**) A549 cells were infected with indicated viruses at an MOI of 2 to measure RNA production. At 8 hpi, RNA was extracted, and vRNA, cRNA, and mRNA of the NP gene were determined by strand-specific qRT-PCR. (**c**) A549 cells were infected with the viruses at an MOI of 2, and NP expression in infected cells was determined by western blot analysis. *N*=3 biological replicates. (**d**) 293T cells were infected with the indicated viruses and transfected with pCAGGS-Cal NP or pCAGGS-mRFP cDNAs. Cell lysates were prepared at 24 hpi, and viral HA, NP, and cellular b-actin were detected by western blotting. The panel indicates the level of HA expression compared with the sample transfected with control pCAGGS-mRFP quantitated by ImageJ. (**e**) 293T cells were infected with Cal 321K/330V virus at an MOI of 1 and transfected with pCAGGS-mRFP (1 μg), pCAGGS-Cal NP (0.5 μg), and pCAGGS-mRFP (0.5 μg) or pCAGGS-Cal NP (1 μg). Total RNA was extracted at 24 hpi, and cRNA of the NP gene was determined by strand-specific qRT-PCR. All error bars show means ± the standard deviations. *N* = 3. Two-way ANOVA followed by Tukey’s multiple comparison test (*P < 0.05, **P < 0.01, ***P < 0.001, ****P < 0.0001).

We previously found that mutations in PA NTD enhance the translation efficiency of viral NP mRNA due to enhanced association of GRSF1 (6). Introduction of T85I and G186S mutations found in Cal PA to Nan PA strongly enhanced the production of NP. Similarly, a single mutation V100I found in Mich PA strongly enhanced translation efficiency of NP mRNA (6). The PA CTD N321K mutation emerged a few years after the introduction of pH1N1 in 2009, and I330V and PA NTD V100I emerged and became dominant a few years later. All three mutations have been fixed in the viral population since then (Fig. S3). With the *in vitro* assay, we found that a higher dose of NP protein increased genome replication (Fig. 2a). Therefore, we anticipated that the increased NP production by the PA V100I mutation in conjunction with the PA CTD mutations is required for enhanced genome replication and growth in virus-infected cells. To test this, we rescued an additional Cal virus containing Mich mutations in both the NTD and CTD at V100I, N321K, and I330V, and virus growth and RNA production were compared with the virus containing only N321K and I330V mutations. Strikingly, the additional V100I mutation restored the attenuated phenotype of the N321K/I330V mutant and grew at similar levels as Cal wt (Fig. 8a). Similarly, genome replication and transcription activities in infected cells were restored with the introduction of the 100I residue (Fig. 8b). We confirmed that the NP production by the Cal N321K/I330V virus was limited compared with the virus containing the additional V100I mutation (Fig. 8c). To further determine the effect of increased NP expression on viral protein production and genome replication, we infected cells with the mutant viruses and transfected them with pCAGGS-Cal NP cDNAs. In Cal N321K/I330V virus-infected cells, viral HA production was indeed enhanced by overexpressing NP (Fig. 8d). Additionally, increased NP expression enhanced genome replication in Cal N321K/I330V-infected cells. Transfection with 0.5 or 1.0 μg of NP cDNA enhanced cRNA levels 21-fold and 26-fold, respectively (Fig. 8e). Together, these results indicate that PA NTD and CTD host-adaptive mutations work synergistically for rapid viral growth.

## DISCUSSION

Multiple host-adaptive mutations are required for avian IAVs to spill over into human populations. Avian IAVs need to mutate their genes to (i) bind the alpha-2,6-SA receptors found in the upper respiratory tract of humans, (ii) utilize host factors, such as ANP32 for genome replication, and (iii) evade innate host restriction factors (8, 35–38). One of the best-characterized host-adaptive mutations is PB2 E627K, which has been present in all pandemic IAVs, except pH1N1. The single mutation of PB2 E627K in avian IAV has been shown to increase activity of the vRdRp in mammalian cells and significantly enhance viral replication and pathogenicity (11, 39). The mechanism of this activation has been a mystery for decades, but recent analysis revealed that the E627K mutation enables the vRdRp to interact with cellular ANP32A/B and enhance efficient viral genome replication (40, 41). Further structural analysis revealed that ANP32 is a key component of the replicase (vRdRp dimer) complex, and PB2 E627K enhances the association of the vRdRp with ANP32 (Fig. 3a). Additionally, this interaction is species-specific, and avian vRdRp, which includes PB2 627E, cannot efficiently use human ANP32 to form the replicase (12, 13). 2009 pH1N1 has PA and PB2 genes originating from avian IAV, but it lacks the PB2 E627K. It is highly likely that during circulation within swine host, the virus obtained mutations in its PB2 or PA or both, which made it infectious to human hosts. The emergence of pH1N1 indicates that PB2 E627K is not essential for IAV to infect humans and alternative adaptive mechanisms exist for IAV fitness to humans.

Our lab has been studying how pH1N1 adapted to humans because understanding the mechanism of host adaptation is crucial for the prevention of future pandemics. Previous work from our lab showed that the 2009 pH1N1 Cal virus PA and PB2 components can independently activate the avian vRdRp (7, 30). Notably, the enhancement of avian vRdRp by Cal PA was much stronger than that of Cal PB2, indicating that mutations in PA by themselves can enhance activity of the avian vRdRp (6, 7). However, these studies were carried out using a luciferase reporter gene in transfected cells, and the readout was the level of luciferase activity produced by the vRdRp. The ability of the vRdRp to replicate viral genomes was not directly determined. Using the reporter assay optimized to measure genome replication directly by qRT-PCR, we compared the impact of Cal PA and PB2 in activating an avian vRdRp. We found that both PA and PB2 strongly increased genome replication activity (Fig. 2c). Several residues of pH1N1 PB2 have been reported to increase replication and viral growth (24). Our lab also reported that Cal PB2 mutation T271A enhanced the overall activity of the Nan vRdRp in mammalian cells and enhanced viral growth in mice (30, 42). Similarly, the Cal PB2 T588I mutation enhanced vRdRp activity in mammalian cells and increased pathogenicity in mice (43). Notably, Cal PB2 mutation L591R has been shown to enhance vRdRp interaction with mammalian ANP32, leading to increased replication (25). Human ANP32 lacks a 33 amino acid insertion present in avian ANP32, which contains a hydrophobic SUMO interaction motif (SIM) (13). Although the acidic glutamic acid at avian IAV PB2 627 can recruit avian ANP32, the basic lysine mutation at human IAV PB2 627 is considered to function better at recruiting human ANP32. Instead of PB2 E627K, Cal PB2 has mutations at 271, 590, and 591 that allow the recruitment of mammalian ANP32 (25). This indicates that host-adaptive mutations in the avian origin Cal PB2 were important to recruit ANP32 and enhance vRdRp activity when pH1N1 initially emerged into human hosts.

We also found that Mich PA can enhance avian vRdRp activity better than Cal PA or even Cal PB2, suggesting that additional PA mutations introduced during seasonal circulation further enhanced genome replication activity. This is supported by the fact that the N321K and I330V mutations specifically enhanced Cal vRdRp cRNA synthesis from vRNA (Fig. 3d). More importantly, the Nan PA containing the key four mutations at the dimer interface (N321K/I330V/L336M/K356R) was as active as the Mich PA in genome replication (Fig. 3c). We also detected enhanced vRdRp oligomer formation by these mutations (Fig. 4 and 5). It is not clear if these mutations are involved in efficient recruitment of ANP32, which is essential for IAV genome replication and stabilization of the replicase (44). However, efficient recruitment of ANP32 is likely not the only mechanism to enhance genome replication. One potential mechanism is that, due to their location, these mutations enhance asymmetric dimer formation independent of ANP32 by stabilizing interactions between the two vRdRp complexes of the asymmetric dimer (45). Enhanced genome replication can then result in faster progeny virion production and viral transmission.

**Fig 9:**
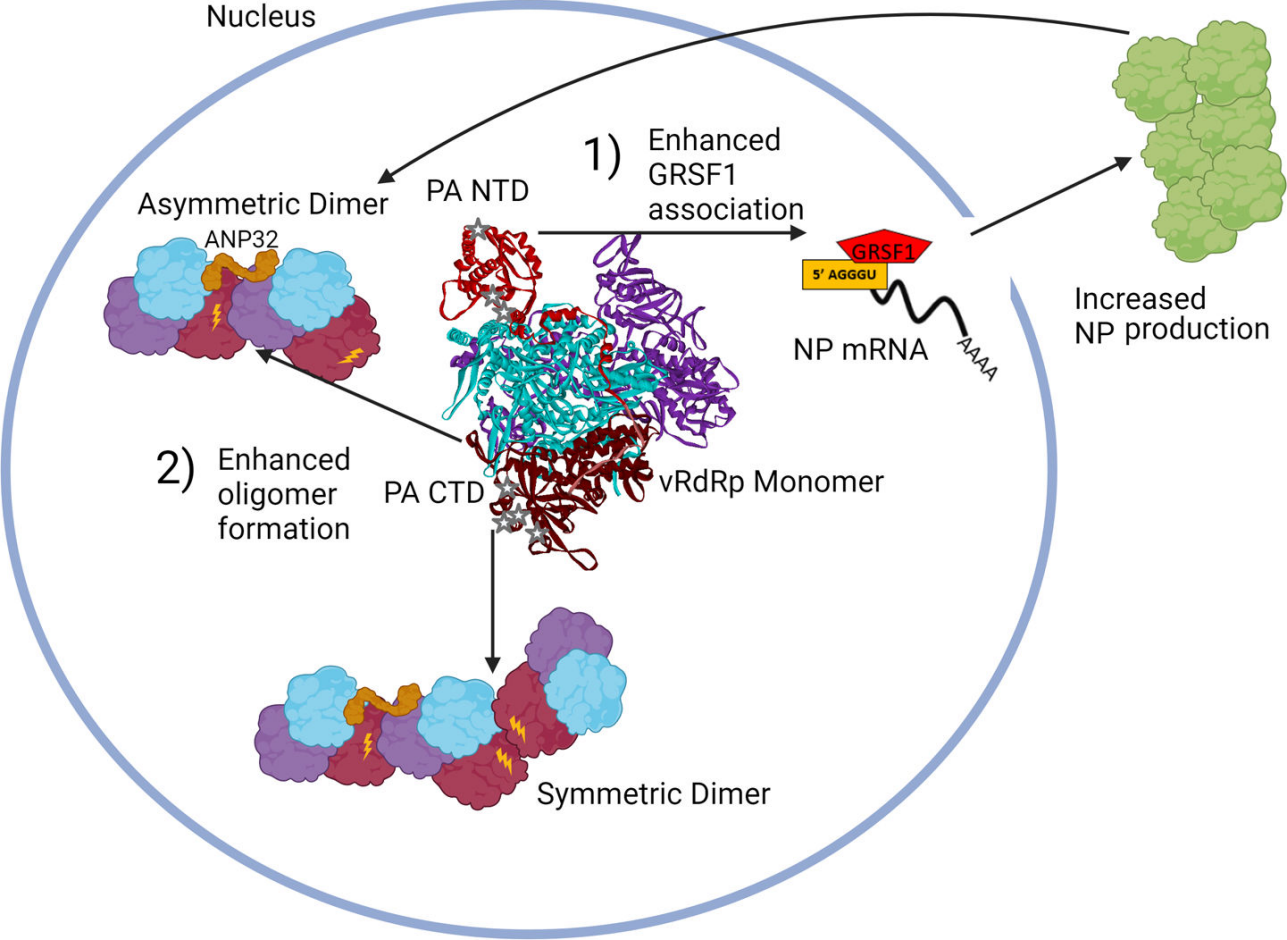
Model of host adaptation by pH1N1 PA. Host--adaptive mutations in PA enhance viral growth through two interdependent mechanisms. (1) PA NTD mutations enhance association of NP mRNA with host protein GRSF1, resulting in increased NP production. (2) Increased NPs encapsidate and protect progeny viral genomes to support enhanced genome replication resulted from the PA CTD mutations. Created in BioRender. Takimoto, T. (2024) https://BioRender.com/w60t796.

Our *in vitro* analysis indicates that the key PA CTD mutations enhance genome replication activity of the vRdRp. However, a mutant pH1N1 virus containing the enhancing PA CTD mutations was attenuated in infected cells (Fig. 6 and 7). These results indicate that enhancing genome replication activity by itself is detrimental for virus growth. This seems to be reasonable because the PA CTD may alter the balance between transcriptase and replicase vRdRp activities in infected cells, reducing the production of NP, which is required for the protection of replicated viral genome. In fact, we found that genome replication activity was enhanced when additional NP was provided *in vitro* (Fig. 2a). Therefore, in the context of viral infection, enhanced NP expression is required to sustain the enhanced level of genomes produced by a vRdRp containing the PA CTD mutations. Without enhanced NP production, elevated viral RNAs produced in cells will not be encapsidated and degrade rapidly. In fact, our data showed that overexpression of NP from cDNAs enhanced the accumulated genome copies produced by the Cal N321K/I330V virus (Fig. 8E). This enhanced NP expression is provided by the PA NTD mutations introduced to pH1N1. We previously showed that PA NTD mutations (T85I and G186S) enhance GRSF1 association to viral NP mRNA and increase NP expression by improving translation efficiency. An additional host-adaptive mutation of pH1N1, V100I, further enhanced expression of NP (6). We also reported that a mutant virus containing mutations at the GRSF1-binding site on NP gene attenuated NP production, genome replication, and virus growth (46). Consistent with the importance of NP expression, introduction of the PA NTD residue V100I rescued NP production and the attenuated virus growth of PA CTD mutant virus (Fig. 8). The V100I mutation was introduced and maintained in the circulating viral population by 2014, around the time when N321K and I330V were dominant. These residues have remained fixed (Fig. S3). These results suggest that subtle adjustments of genome replication activity and NP expression are required for the virus to enhance growth in a specific host. Adaptive mutations introduced to pH1N1 PA therefore present an alternative mechanism for the virus to accelerate its growth in infected cells. We propose a new model for IAV host adaptation utilized by pH1N1 (Fig. 9). PA CTD mutations enhance genome replication activity by increasing vRdRp oligomer formation, and PA NTD mutations recruit GRSF1 to NP mRNA to increase NP production required for enhanced genome replication. Both activities, induced by PA CTD and NTD mutations, are required for the rapid and enhanced genome replication and virus production in humans. As more of the population acquires immunity to the circulating virus, mutations that accelerate growth and transmission before the virus is recognized and cleared by the immune system are essential for the seasonal circulation and epidemics of IAV (47). Therefore, we expect PA NTD and CTD mutations that enhance viral growth and transmission have been selected for during seasonal circulation. Although Cal PB2 plays a role in host adaptation, our study highlights an unrecognized ability for PA mutations to enhance vRdRp oligomer formation and increase genome replication.

## MATERIALS AND METHODS

### Cell lines and plasmids

MDCK (ATCC: CCL-34), A549 (ATCC: CRM-CCL-185), and 293T (ATCC: CRL-3216) cells were maintained in Dulbecco’s-modified Eagle’s medium (DMEM, Gibco) supplemented with 8% FB Essence (Avantor), 25 mM HEPES (Gibco), and 50 μg/mL gentamicin (Gibco). Nan, Cal, and Mich PA, PB1, PB2, and NP were cloned into pCAGGS and pPolI expression vectors using restriction enzymes as described previously (6, 30). Nan PA L336M was created previously (7). All other Nan PA with host-adaptive mutations and Cal PA with host-adaptive mutations cDNAs were generated using overlapping site-directed mutagenesis. The negative-sense pPolI-NPLuc construct, which contains Firefly Luciferase under the control of the human RNA polymerase I promoter, was obtained from T. Wolff (Robert-Koch Institute, Berlin, Germany). The positive-sense pPolI-NPLuc construct was created by reversing the orientation of the negative-sense pPolI-NPLuc when inserted into the pPolI expression vector. pCAGGS Cal PB2 FLAG tagged, Cal PB2 HA tagged, Nan PB2 FLAG tagged, and Nan PB2 HA-tagged cDNAs were created by amplifying the coding region using a primer at the C-terminal end containing the FLAG or HA sequence and subcloning into empty pCAGGS. pCAGGS-mRFP was created by subcloning the monomeric red fluorescent protein (mRFP) gene from mRFP-C1 (Addgene) to pCAGGS. All plasmids were confirmed by sequencing.

### Transfection-based reporter gene assay

293T cells in 12-well plates were transfected with varying concentrations of vRdRp components and NP along with 0.1 μg of the indicated sense pPolI-NP-luc reporter using Lipofectamine 2000 (Invitrogen) in Opti-MEM (Gibco). In Fig. 1, cells were transfected with 0.4 μg of pCAGGS plasmids containing PA, PB1, PB2, and NP for 16 h at 37 °C. Luciferase activity was measured using the dual-luciferase reporter assay system (Promega) according to the manufacturer’s protocol. In Fig. 2A, the cells were transfected with 40 ng of pCAGGS plasmids containing PA, PB1, PB2, and indicated amount of NP for 16 h at 37 °C. In Fig. 2b through d, 3c and d, the cells were transfected with 40 ng of pCAGGS plasmids containing PA, PB1, PB2, and 2 μg NP for 16 h at 37 °C. In Fig. 3e, the cells were transfected with 40 ng of pCAGGS plasmids containing PA, PB1, PB2, and 2 μg NP for 8 h at 37 °C. Cell lysates were treated with TRIzol Reagent (Invitrogen), and RNA was quantified as described below.

### Quantification of mRNA, cRNA, and vRNA

For measuring NPLuc mRNA, cRNA, and vRNA, 293T cells were transfected as described above. Total RNA was extracted using TRIzol Reagent (Invitrogen) according to the manufacturer’s protocol. RNA concentration and purity were determined with a Nanodrop 2000 (ThermoFisher). One hundred nanograms of purified RNA was used for qRT-PCR with the hot-start modification using RevertAid Reverse Transcription Kit (ThermoFisher). Primers used for RT and qPCR were designed to be strand specific for all three NPLuc RNA species using tags unrelated to IAV and validated as described by Kawakami et al. (28). cDNAs were generated from RNA with a primer that annealed to a unique feature of each RNA (NPLuc mRNA RT Tag: CCAGATCGTTCGAGTCGTTTTTTTTTTTTTTTTTCTTTACAATTT, NPLuc cRNA RT tag: GCTAGCTTCAGCTAGGCATCAGTAGAAACAAGGGTATTTTTCTTTAC, or NPLuc vRNA RT Tag: GGCCGTCATGGTGGCGAATGCAAGGATATGGGCTCAC. Real-time PCR was carried out using SYBR Green PCR Master Mix (Applied Biosystems) with an Applied Biosystems QuantStudio 3 Real-time PCR system. qPCR primers were specific to NPLuc mRNA (Luc 1511F: GTCAAGTAACAACCGCGAAA and mRNA tag R: CCAGATCGTTCGAGTCG), cRNA (Luc 1511F and cRNA tag R: GCTAGCTTCAGCTAGGCATC) or vRNA (NPLuc 1170R: TCCACAACCTTCGCTTCAAA and vRNA tag F: GGCCGTCATGGTGGCGAAT). DNA standards for NPLuc mRNA, cRNA, and vRNA were generated by PCR. A standard curve from 10^9^ to 10^1^ copies was used for quantification.

For measuring viral Cal NP or WSN NA mRNA, cRNA, and vRNA in Fig. 6b, 7c, and 8b, A549 cells were infected with indicated viruses at the MOI indicated in figure legends for 1 h and cultured for 8 h or 16 h at 37 °C. Total RNAs were extracted, and one hundred nanograms of purified RNA was used for RT-PCR as above. Primers used for RT-PCR and qPCR were designed to be strand specific for all three NP or NA RNA species. Primers used for the qRT-PCR are as follows. Cal NP mRNA RT Tag: CCAGATCGTTCGAGTCGTTTTTTTTTTTTTTTTTCCTCAACTGTC, Cal NP cRNA RT tag: GGATCCTAATACGACTCACTATAGGGGTAGAAACAAGGGTATTTTTCCT, Cal NP vRNA RT Tag: GGCCGTCATGGTGGCGAATAAATGGACGAAGGACAAGGGTTGC, WSN NA mRNA RT Tag: CCAGATCGTTCGAGTCGTTTTTTTTTTTTTTTTTGAACAAACTAC, WSN NA cRNA RT Tag: GCTAGCTTCAGCTAGGCATCAGTAGAAACAAGGAGTTTTTTGAAC, and WSN NA vRNA RT Tag: GGCCGTCATGGTGGCGAATACCATAATGACCGATGGCCCAAGT. qPCR primers used for qPCR are as follows. For Cal NP mRNA, 1466F: CGATCGTGCCTTCCTTTG and mRNA Tag R: CCAGATCGTTCGAGTCG. For WSN NA mRNA, 1314F: TGAATAGTGATACTGTAGATTGGTCT and mRNA Tag R. For cRNAs, Cal NP 1466F: CGATCGTGCCTTCCTTTG or WSN NA 1314F: TGAATAGTGATACTGTAGATTGGTCT and cRNA Tag R: GCTAGCTTCAGCTAGGCATC. For vRNAs, Cal NP 846R: CCTCAGAATGAGTGCTGACCGT or WSN NA 839R: ACATCACTTTGCCGGTATCAGGGT and vRNA Tag F: GGCCGTCATGGTGGCGAAT. DNA standards for Cal NP and WSN NA mRNA, cRNA, and vRNA were generated by PCR. A standard curve from 10^9^ to 10^1^ copies was used for quantification.

For Fig. 8e, 293T cells in a 12-well plate were infected with Cal N321K/I330V virus at an MOI of 1 for 1 h and then transfected with pCAGGS-mRFP (1 μg), pCAGGS-Cal NP (0.5 μg), and pCAGGS-mRFP (0.5 μg) or pCAGGS-Cal NP (1 μg) using Lipofectamine 2000 (3 μL). Total RNA was extracted at 24 hpi, and cRNA of the NP gene was determined by strand-specific qRT-PCR as described above.

### Sequence analysis

Sequences of the polymerase genes of A/California/04/2009(H1N1), A/Michigan/272/2017(H1N1), and A/Chicken/Nanchang/3-120/01(H3N2) were obtained from fludb.org and correspond to NCBI:txid641501, NCBI:txid2033552, and NCBI:txid215853.

### Analysis of RdRp oligomer formation by immunoprecipitation

For the analysis of Cal RdRps, 293T cells in 6-well plates were transfected with 600 ng of pCAGGS Cal PB1, 600 ng of Cal or Mich PA, and 300 ng each of untagged, Flag- or HA-tagged Cal PB2 as indicated by Lipofectamine 2000 in Opti-MEM and incubated at 37 °C for 24 h. Cal and Mich PAs used in this experiment contain a mutation at the endonuclease active site (K134A) to exclude the effect of PA-X shutoff activity (48). Cells were then lysed with 400 μL of RIPA lysis buffer (25 mM Tris-HCl pH 8.0, 150 mM NaCl, 1% NP-40, 1% Triton X-100, 0.5% Sodium Deoxycholate, 0.1% SDS) containing Halt protease inhibitor (PI87785, ThermoFisher), sonicated, and centrifuged for 10 min at 10,000 × *g* at 4 °C. Supernatant (200 μL) was used for immunoprecipitation. A half milligram of Protein G Dynabeads (Invitrogen) was incubated with 2 μg rabbit anti-FLAG (D6W5B, Cell Signaling) per sample for 2 h at room temperature while rotating. The Ab-Dynabes complex was then incubated with the lysate mixture and incubated for 16 h at 4 °C while rotating. The associated proteins were eluted from Dynabeads using 1× NuPAGE LDS sample buffer. For the analysis of Nan RdRps, cells were transfected with cDNAs expressing indicated proteins together using Lipofectamine. The cell lysates were prepared as above and used for the pull-down assay.

### RdRp oligomer formation analyzed by sucrose gradient fractionation

293T cells in 10 cm dishes were transfected with pCAGGS plasmids of 5 μg Nan PB1, 5 μg indicated Nan PA, 2.5 μg each Nan PB2-FLAG, and Nan PB2-HA by Lipofectamine 2000 in Opti-MEM. Lipofectamine was replaced with cell growth media after 8 h post-transfection, and the cells were incubated at 37 °C for 36 h. Cells were then lysed in 800 μL of RIPA lysis buffer containing Halt protease inhibitor, sonicated, and cleared by centrifugation. A total of 750 μL lysate was loaded on top of a 10%–40% linear sucrose gradient prepared in high salt lysis buffer (5M NaCl, 1M Tris-HCl pH 7.5, 1M MgCl_2_) and centrifuged for 90 min at 39,000 rpm at 4 °C using a Beckman Coulter SW 41Ti rotor (49). Gradients were fractionated using a BR-188 Density Gradient Fractionation System (Brandel). Gradients were separated into 18 fractions, and protein expression was measured using western blot assays as described below.

### Western blotting analysis

For immunoblotting, cell lysates were resolved by SDS-PAGE using 10% acrylamide gels and transferred to 0.45 μM PVDF membranes. For the detection of protein, the following primary antibodies were used: rabbit anti-FLAG tag (1:500 dilution, ab1162, abcam), mouse anti-HA tag (1:1,000 dilution, 26183, Invitrogen), mouse anti-PA (1:1,000 dilution, F5-32 (48)), mouse anti-PB1 (1:500 dilution, F5-10 (50)), rabbit polyclonal anti-PB2 (1:1,000 dilution, GTX125926, GeneTex), rabbit polyclonal anti-PA (1:1,000 dilution, GTX125932, GeneTex), rabbit polyclonal anti-PB1 (1:1,000 dilution, GTX125923, GeneTex), rabbit anti-NP (1:3,000 dilution, GTX125989, GeneTex), mouse monoclonal anti-HA (1:3,000 dilution, NR-28666, BEI resources), and mouse anti-β-actin (1:6,000 dilution, 8H10D10, Cell Signaling Technology). Secondary antibodies used were horse anti-mouse IgG HRP-linked (1:3,000 dilution, 7076, Cell Signaling Technology), goat anti-rabbit IgG HRP-linked (1:3,000 dilution, 7074, Cell Signaling Technology) and goat anti-mouse IgG1 HRP-linked (1:3,000 dilution, ab97240, abcam). Samples were visualized using SuperSignal™ West Dura Extended Duration Substrate (ThermoFisher) with a BioRad Chemidoc XRS Imager. Pixel density of the images was measured using ImageJ software. For Fig. 4b, immunoprecipitated HA band intensity for three independent replicates was normalized to that of the corresponding immunoprecipitated FLAG band intensity for each sample and then shown as relative to WT Nan PA. For Fig. 5, quantities of PB2-HA in each fraction were measured, and total quantities were calculated. The percentage of the proteins in each fraction was then calculated. For Fig. 8d, 293T cells in a 12-well plate infected with mock or mutant viruses were transfected with 0.5 μg of cDNAs expressing Cal NP or mRFP. Quantities of HA in the presence of overexpressed NP were compared with that overexpressing control mRFP samples.

### Viruses and viral infections

Wild-type and recombinant Cal or WSN with Nan polymerase components containing host-adaptive mutations were rescued in 293T/MDCK co-cultured cells (6, 7, 51). Rescued viruses were plaque purified and sequenced. Viral infections were carried out in DMEM containing 0.15% bovine serum albumin (DMEM-BSA), 25 mM HEPES (Gibco), and 50 μg/mL gentamicin (Gibco). For single-cycle virus growth and RNA quantification, A549 cells were infected with viruses at an MOI of 3, 2, or 0.2 as indicated for 1 h at 37°C and cultured in the same medium. At specific time points post-infection, supernatants were collected and treated with acetylated trypsin at the final concentration of 2 μg/mL for 1 h at 37°C. The virus titers were then measured by TCID_50_ in MDCK cells in the medium containing acetylated trypsin at 2 mg/mL. At 8 h post-infection, cells were treated with TRIzol Reagent (Invitrogen), and RNA was extracted according to the manufacturer’s protocol. RNA quantification by strand-specific qRT-PCR was carried out as described above.

## Data Availability

All study data are included in the article.

## SUPPLEMENTAL MATERIAL

Fig. S1 jvi.01391-25-s0001.tif PA and PB2 residues that differ between the viruses used in this study.

Fig. S2 jvi.01391-25-s0002.tif Reporter cDNAs expressing positive sense and negative sense template RNAs.

Fig. S3 jvi.01391-25-s0003.tif Emergence of PA mutations at residues 100, 321, and 330 by year.

Fig. S4 jvi.01391-25-s0004.tif Location of the C-terminal region of PB2 where HA or Flag tag was fused.

ASM does not own the copyrights to Supplemental Material that may be linked to, or accessed through, an article. The authors have granted ASM a non-exclusive, world-wide license to publish the Supplemental Material files. Please contact the corresponding author directly for reuse.
