# Supplementary figures and images for: Enhanced genome replication activity of pandemic H1N1 influenza A virus through PA mutations
Source: J Virol. 2025 Dec 23;100(2):e01391-25. doi: 10.1128/jvi.01391-25 (PMC12911886; doi:10.1128/jvi.01391-25)

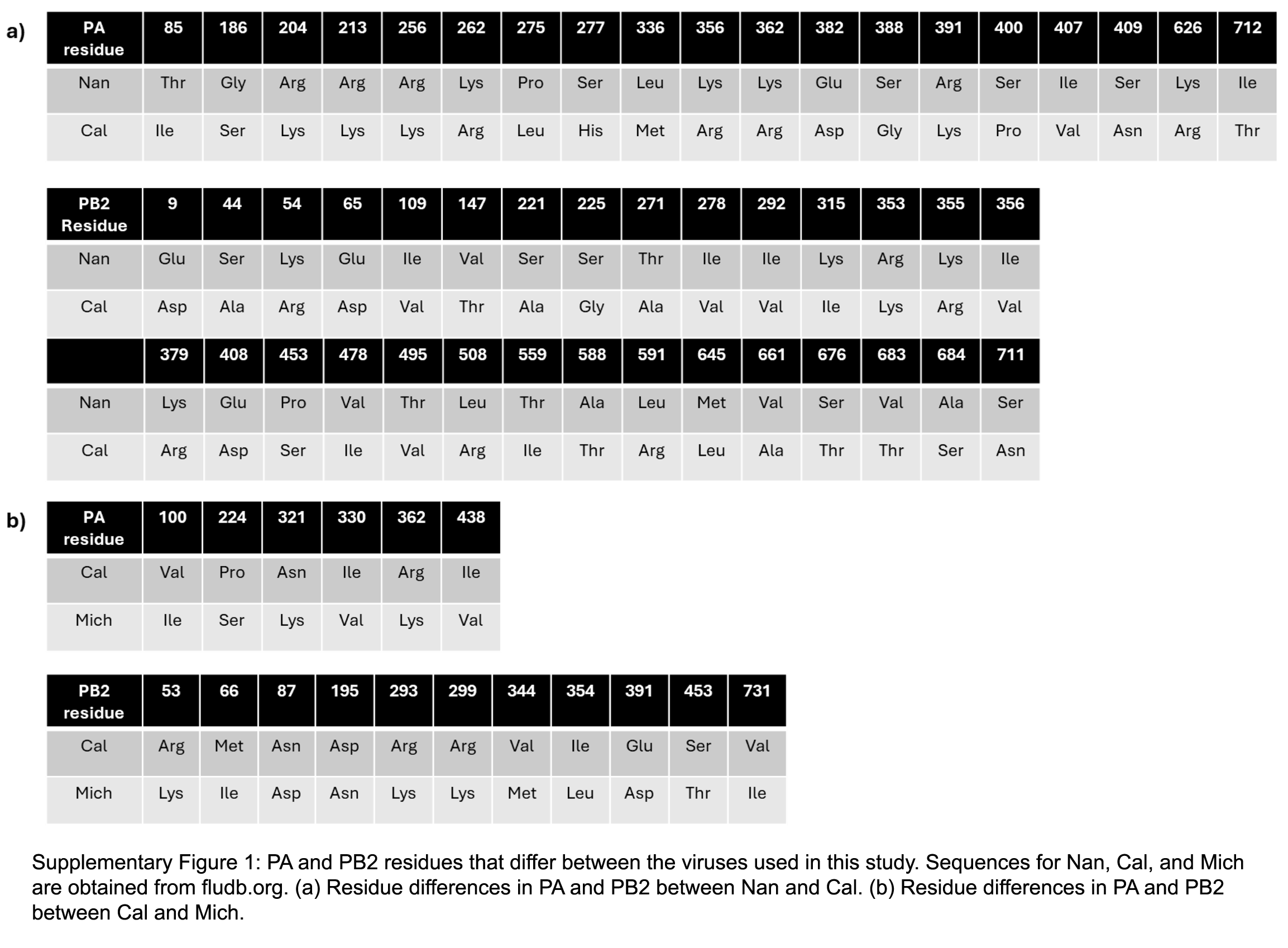

Supplement: Fig. S1 — PA and PB2 residues that differ between the viruses used in this study. [file jvi.01391-25-s0001.tif]

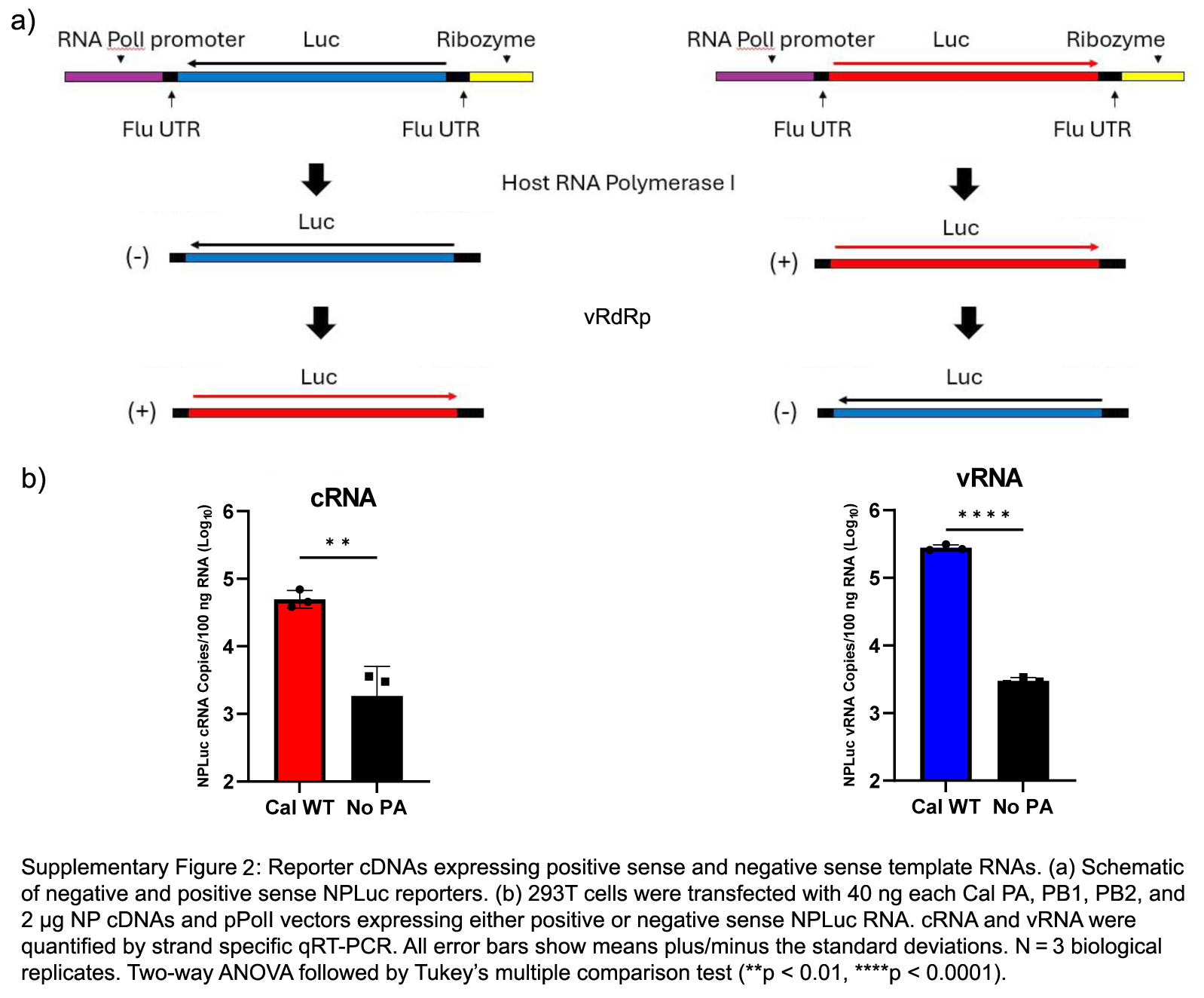

Supplement: Fig. S2 — Reporter cDNAs expressing positive sense and negative sense template RNAs. [file jvi.01391-25-s0002.tif]

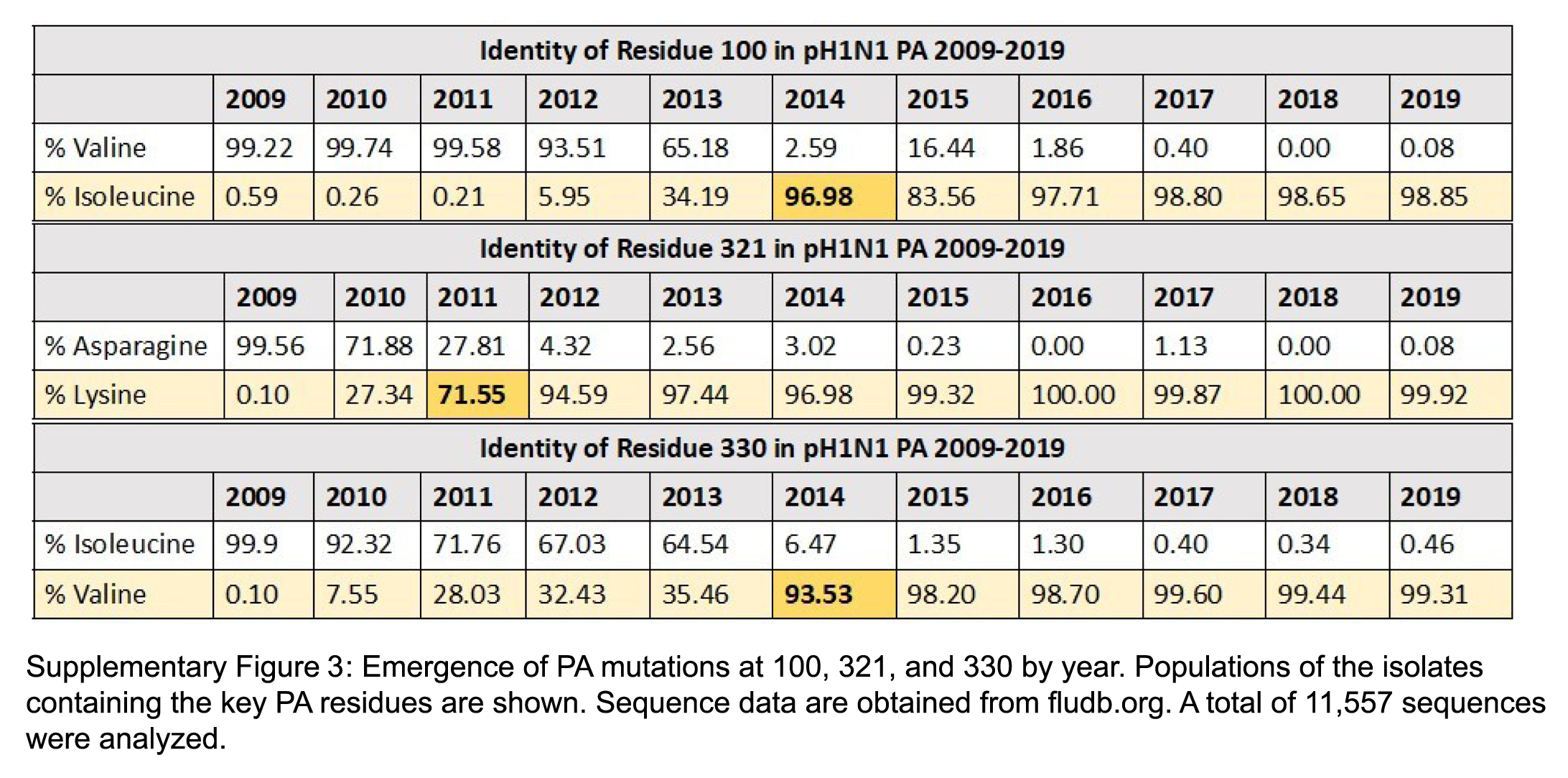

Supplement: Fig. S3 — Emergence of PA mutations at residues 100, 321, and 330 by year. [file jvi.01391-25-s0003.tif]

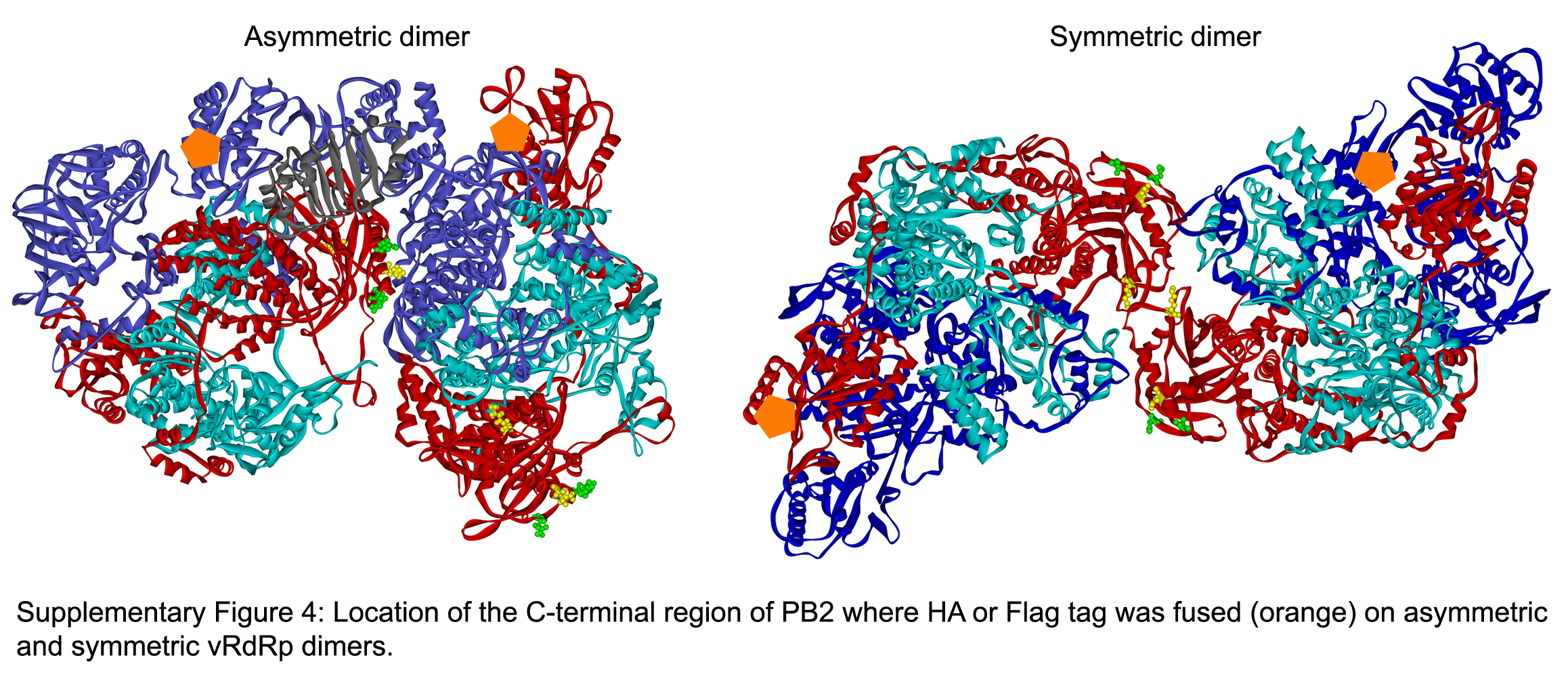

Supplement: Fig. S4 — Location of the C-terminal region of PB2 where HA or Flag tag was fused. [file jvi.01391-25-s0004.tif]
